# Clinical Genetic Testing in Autism Spectrum Disorder in a Large Community-Based Population Sample

**DOI:** 10.1001/jamapsychiatry.2020.0950

**Published:** 2020-05-13

**Authors:** Daniel Moreno-De-Luca, Brian C. Kavanaugh, Carrie R. Best, Stephen J. Sheinkopf, Chanika Phornphutkul, Eric M. Morrow

**Affiliations:** 1 Division of Child and Adolescent Psychiatry, Department of Psychiatry and Human Behavior, Warren Alpert Medical School of Brown University, Providence, Rhode Island; 2 Hassenfeld Child Health Innovation Institute, Brown University, Providence, Rhode Island; 3 Emma Pendleton Bradley Hospital, East Providence, Rhode Island; 4 Center for Translational Neuroscience, Robert J. and Nancy D. Carney Institute for Brain Science and Brown Institute for Translational Science, Brown University, Providence, Rhode Island; 5 Department of Pediatrics, Warren Alpert Medical School of Brown University, Providence, Rhode Island; 6 Brown Center for the Study of Children at Risk, Women and Infants Hospital of Rhode Island, Providence; 7 Division of Human Genetics, Warren Alpert Medical School of Brown University, Rhode Island Hospital, Providence; 8 Department of Molecular Biology, Cell Biology and Biochemistry, Brown University, Providence, Rhode Island

## Abstract

This study analyzes data from a large, population-based study of people with autism spectrum disorder to evaluate what proportion underwent genetic testing.

Autism spectrum disorder (ASD) is among the most strongly genetic neuropsychiatric conditions, with an increased frequency of rare, deleterious copy number variants and single-nucleotide variants. Because of this, several medical professional societies have recommended offering chromosomal microarray (CMA) testing and Fragile X testing for people with ASD,^1^ with growing support for exome sequencing as the first-tier genetic test.^2^ To understand the implementation of genetic testing in a real-world population, we analyzed data from the Rhode Island Consortium for Autism Research and Treatment (RI-CART) study, a large, population-based study of people with ASD.^3^

## Methods

This study was approved by the institutional review board at Lifespan, and all participants provided written informed consent. We analyzed self-report data and medical records, when available, from 1280 participants in the RI-CART study, recruited between April 1, 2013, and April 30, 2019, with ASD diagnosis confirmed by assessment using the Autism Diagnostic Observation Schedule, Second Edition (ADOS-2).^3^ Statistical analyses included Pearson correlations, χ^2^ analyses, and analyses of variance. Statistical significance was set at a 2-sided *P *value less than .05.

## Results

Of these 1280 participants with confirmed ASD diagnosis by ADOS-2, ages ranged from 1.75 years to 68.48 years, and 16.5% (n = 211) reported having received some genetic testing, as follows: Fragile X in 13.2% (n = 169), karyotype in 7.2% (n = 92), and CMA in 4.5% (n = 57). Remarkably, only 3% of participants (n = 39) reported having received both recommended tests (Fragile X and CMA); 9.4% (n = 121) reported that they were unsure whether they had received any testing; and 21.4% did not answer (n = 274).

We next examined factors associated with receiving genetic testing. Participants who reported any genetic testing showed an earlier age at ASD diagnosis (mean age, 4.2 years; range, 1.33-27.1 vs 6.1 years; range, 1.2-51.0; *F*_1,597_ = 13.258; *P* < .001), greater ASD severity (mean [SD] ADOS-2, 7.33 [1.8] vs 6.99 [1.8]; *F*_1,1169_ = 5.583; *P* = .02), and higher frequency of intellectual disability (odds ratio, 3.327; 95% CI, 2.382-4.649; *P* < .001) and epilepsy (odds ratio, 3.093; 95% CI, 1.748-5.474; *P* < .001).

We examined factors associated specifically with CMA testing (Table). Patients diagnosed by subspecialist pediatricians were more likely to report genetic testing compared with those diagnosed by psychiatrists and psychologists. Analysis by age at enrollment indicated that younger participants were more likely to report having received CMA testing (Figure, A). Analysis by calendar year of ASD diagnosis indicated that CMA testing increased, and Fragile X and karyotype testing decreased in the last decade (Figure, B). These results reflect changes in genetic testing practices; however, a sustained overall low frequency of genetic testing in the group remains.

**Table.** Clinical Factors Associated With CMA Testing^a^

Characteristic	No.^b^	No. (%)		*F*/χ^2^	*P* value
		CMA (n = 57)	No CMA (n = 815)		
Clinical presentation					
Male	872	40 (70.2)	650 (79.8)	2.960	.09
Age at enrollment, mean (SD), y	872	9.1 (5.3)	12.8 (9.3)	8.758	.003
Verbal yes (parent report)^c^^,^^d^	840	40 (70.2)	706 (90.2)	21.365	<.001
ADOS-2 severity, mean (SD)^e^	808	7.2 (1.7)	7.0 (1.8)	.769	.38
VABS-II ABC, mean (SD)	666	68.8 (18.0)	73.0 (17.7)	2.198	.14
ASD diagnosis					
Age at ASD diagnosis, mean (SD), y	423	4.5 (3.5)	6.0 (6.0)	2.325	.13
ASD diagnosing clinician^f^^,^^g^					
Pediatrician	431	13 (41.9)	57 (14.3)	18.062	<.001
Psychiatrist	431	5 (16.1)	85 (21.3)		
Psychologist	431	6 (19.4)	175 (43.8)		
Neurologist	431	7 (22.6)	83 (20.8)		
Co-occurring diagnoses					
Epilepsy	872	4 (7)	30 (3.7)	1.583	.21
Intellectual disability	872	15 (26.3)	109 (13.4)	7.315	.007
Demographics					
Private insurance	872	37 (65)	519 (63.7)	.035	.85
Medicaid insurance	872	41 (72.0)	384 (47.1)	13.129	<.001

Abbreviations: ADOS-2, Autism Diagnostic Observation Schedule, Second Edition; ASD, autism spectrum disorder; CMA, chromosomal microarray; VABS-II ABC, Vineland Adaptive Behavior Scales, Second Edition: Adaptive Behavior Composite.

^a^ Results reflect a subsample of the total sample (n = 1280) that indicated yes or no to prior CMA testing (n = 872).

^b^ Variables with a number less than 872 reflect data not completed by participants.

^c^ Verbal yes: participant is able to verbally communicate, based on parent report.

^d^ Reported data reflect a denominator of 57 for CMA and of 783 for no CMA.

^e^ A severity score was not available for 64 participants, resulting in n = 808.

^f^ ASD diagnosing clinician: type of clinician who diagnosed the participant with ASD.

^g^ Reported data reflect a denominator of 31 for CMA and of 400 for no CMA.

**Figure.**
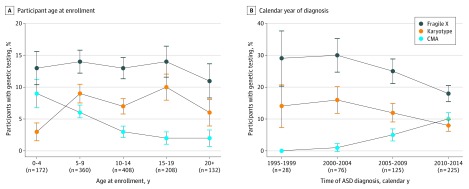
Percentage of Rhode Island Consortium for Autism Research and Treatment (RI-CART) Study Participants With Genetic Testing A, Percentage of participants who reported genetic testing with respect to the age of the participant at the time of enrollment in the RI-CART study between 2013 and 2019. Participants aged 10 years and older have a very low rate of the most modern tests such as chromosomal microarray. B, Percentage of participants who reported genetic testing with respect to the calendar year in which the participant received an autism spectrum disorder diagnosis. Error bars indicate 95% CI.

## Discussion

This study shows that only 3% of participants reported having undergone the recommended clinical genetic testing for ASD, highlighting a dissonance between professional recommendations and clinical practice. Multiple possible reasons exist for this gap, including (1) participant preferences, although current evidence shows that most parents of people with ASD have favorable attitudes toward genetic testing^4,5^; and (2) insurance coverage constraints,^5^ but this has changed after the appearance of medical professional recommendations. Interestingly, we see no difference in CMA testing in participants with and without private insurance but a strong increase in testing in participants with public insurance (Table). Other reasons include (3) limits in clinician knowledge and comfort with genetic testing, with our data showing a lower frequency of genetic testing in people diagnosed with ASD by psychiatrists and psychologists and (4) changes in genetic testing practices over time and a reduced likelihood of adults with ASD being offered testing. Study limitations include that these results were based largely on participant self-report. In conclusion and moving forward, addressing the barriers to testing is crucial to enhance the implementation of genetic testing in clinical practice so that every person with ASD can receive optimal care.
